# Simultaneous detection of Human Immunodeficiency Virus 1 and Hepatitis B virus infections using a dual-label time-resolved fluorometric assay

**DOI:** 10.1186/1477-3155-8-27

**Published:** 2010-11-26

**Authors:** Tiina Myyryläinen, Sheikh M Talha, Sathyamangalam Swaminathan, Raija Vainionpää, Tero Soukka, Navin Khanna, Kim Pettersson

**Affiliations:** 1 Department of Biotechnology, University of Turku, Turku, Finland; 2 Recombinant Gene Products Group, International Centre for Genetic Engineering & Biotechnology, Aruna Asaf Ali Marg, New Delhi-110067, India; 3 Department of Virology, University of Turku, Turku, Finland

## Abstract

A highly specific and novel dual-label time-resolved immunofluorometric assay was developed exploiting the unique emission wavelengths of the intrinsically fluorescent terbium (Tb^3+^) and europium (Eu^3+^) tracers for the simultaneous detection of human immunodeficiency virus 1 (HIV-1) and hepatitis B virus (HBV) infections, respectively. HIV-1 infection was detected using a double antigen sandwich format wherein anti-HIV-1 antibodies were captured using an *in vivo *biotinylated version of a chimeric HIV-1 antigen and revealed using the same antigen labeled with Tb^3+ ^chelate. Hepatitis B surface antigen (HBsAg), which served as the marker of HBV infection, was detected in a double antibody sandwich using two monoclonal antibodies (mAbs), one chemically biotinylated to capture, and the other labeled with Eu^3+ ^nanoparticles, to reveal. The performance of the assay was evaluated using a collection (n = 60) of in-house and commercially available human sera panels. This evaluation showed the dual-label assay to possess high degrees of specificity and sensitivity, comparable to those of commercially available, single analyte-specific kits for the detection of HBsAg antigen and anti-HIV antibodies. This work demonstrates the feasibility of developing a potentially time- and resource-saving multiplex assay for screening serum samples for multiple infections in a blood bank setting.

## Findings

The World Health Organization recommends screening for infections by human immunodeficiency virus (HIV), hepatitis B virus (HBV), hepatitis C virus (HCV) and *Treponema pallidum *(syphilis) for the provision of a safe blood supply [1]. Currently these infections are detected using independent tests. In a step towards a multiplex assay for blood bank screening, we have explored the feasibility of developing an integrated dual-label assay designed to identify infections by HIV and HBV.

**Figure 1:**
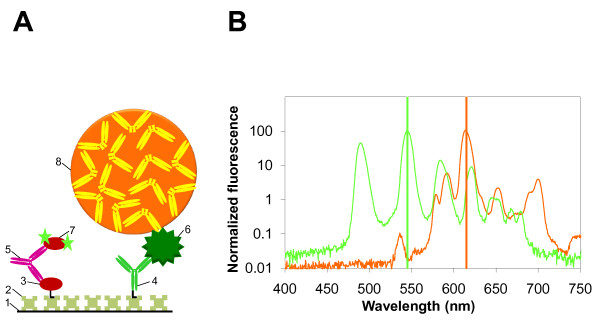
**Design of the dual-label time-resolved immunofluorometric assay**. (A) A schematic illustration of the assay for simultaneous detection of HIV and HBV infections. The Arabic numerals indicate individual assay components: (1) microtitre well surface; (2) streptavidin; (3) r-Bio-HIV-1 Env; (4) Bio mAb 21B; (5) anti-HIV-1 antibodies in infected serum; (6) HBsAg in infected serum; (7) r-HIV-1env labeled with Tb^3+ ^chelate (which is measured at 545 nm); (8) 5 S F(ab)_2 _coated Eu^3+ ^nanoparticles (which is measured at 615 nm). (B) The emission spectra of Tb^3+ ^chelate (green line) and Eu^3+ ^nanoparticles (orange line).

We have exploited the inherent fluorescence of lanthanide chelates to develop a screening assay for the simultaneous detection of HIV and HBV infections based on time resolved fluorometry (TRF) of terbium (Tb^3+^) and europium (Eu^3+^) labels. TRF technology using lanthanide chelates with high fluorescence intensity coupled to very low background signals, made possible by the temporal separation of long-lived emission signals, has the potential for achieving very high levels of sensitivity [2-5]. Consequently, lanthanide chelate-based TRF assays are available commercially for the detection of hormones, tumor markers, celiac disease markers and for neonatal screening. A recombinant HIV-1 env (r-HIV-1env) antigen and two HBsAg specific monoclonal antibodies (mAbs), 21B and 5 S, were created first (unpublished data). The principle of the dual-label TRF assay is depicted pictorially in Figure 1A. Serum analytes were captured efficiently using specific biotinylated binders immobilized at high density on streptavidin (SA)-coated plates. We used an *in vivo *biotinylated version of the r-HIV-1 env protein (r-Bio-HIV-1 env) and chemically biotinylated mAb 21B (Bio-mAb 21B), immobilized on SA-coated microtiter wells, to capture anti-HIV-1 antibodies and HBsAg, respectively. Captured anti-HIV-1 antibodies were detected with Tb^3+ ^chelate-labeled r-HIV-1env antigen. For the detection of captured HBsAg, we utilized the F(ab)_2 _fragment of 5 S mAb. The Fc portion of the antibody molecule can frequently give rise to falsely positive or negative results through interaction with other reagents of the test or normal constituents of patient samples. Its elimination enzymatically or through recombinant expression of antibody fragment has been shown to significantly decrease this source of error [6,7]. Therefore, we cleaved 5 S mAb with bromelain to produce 5 S F(ab)_2 _fragment, and covalently coupled it to carboxyl-activated Fluoro-Max™polystyrene nanoparticles, doped with Eu^3+ ^chelate and used it as the tracer to detect HBsAg. In contrast to Tb^3+^, Eu^3+ ^is available commercially in a nanoparticle format, which has been shown to improve the detection sensitivity greatly [8-10]. The TRF assay described here differs from those reported earlier. It utilizes labels that provide optimal fluorescence without the need for a separate dissociation-based fluorescence enhancement of the DELFIA assays [2,4,5] or a non-dissociative signal development step of the LANFIA procedure [3] and permits measurement of the fluorescence directly from the dry surface of the microtiter wells.

**Figure 2:**
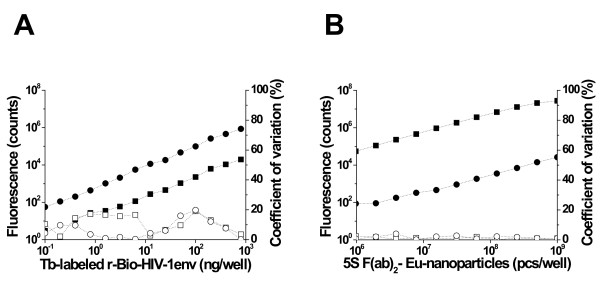
**Cross-talk between the two lanthanide labels used in the assay**. (A) Tb^3+ ^cross-talk. (B) Eu^3+ ^cross-talk. The filled symbols represent the fluorescence and the empty symbols represent the co-efficient of variation, with circles and squares representing data points pertaining to Tb^3+ ^and Eu^3+^, respectively.

We first evaluated the potential for cross-talk between the two fluorescent labels. The emission spectra of Eu^3+ ^chelate-doped nanoparticles and Tb^3+ ^chelate, recorded using a Cary Eclipse spectrofluorometer (Varian, USA) are shown in Figure 1B. The data show that while Eu^3+ ^fluorescence at the emission maximum of Tb^3+ ^is negligible (< 0.02% at 545 nm), Tb^3+ ^fluorescence at 615 nm, the emission maximum of the Eu^3+^, was almost 3%. In order to determine the magnitude of this cross-talk in the actual assay setting, a dilution series of Tb^3+ ^labeled r-Bio-HIV-1env was immobilized on to SA-coated microtiter wells, followed by washing and measurement of fluorescence, using a Victor 1420 multilabel counter (Perkin Elmer Life and Analytical Sciences, Singapore), at 545 nm and 615 nm. The results of this experiment, shown in Figure 2A, indicate the magnitude of Tb^3+ ^cross-talk one may expect while measuring Eu^3+ ^fluorescence at 615 nm in a dual-label assay. Depending on the instrument used, Tb^3+ ^cross-talk was determined to range from 1.2-2.5%. Thus, all Eu^3+ ^fluorescence data were corrected using the measuring instrument-specific Tb^3+ ^cross-talk. Eu^3+ ^cross-talk in Tb^3+ ^fluorescence measurement was determined as shown in Figure 2B. In this experiment, chemically biotinylated rHBsAg was immobilized on SA-coated microtiter wells, and incubated with a dilution series of Eu^3+ ^doped 5 S F(ab)_2 _nanoparticles. As before, fluorescence was measured at both wavelengths (545 nm and 615 nm). The results showed that Eu^3+ ^is unlikely to manifest significant cross-talk during the measurement of Tb^3+ ^fluorescence at 545 nm.

**Figure 3:**
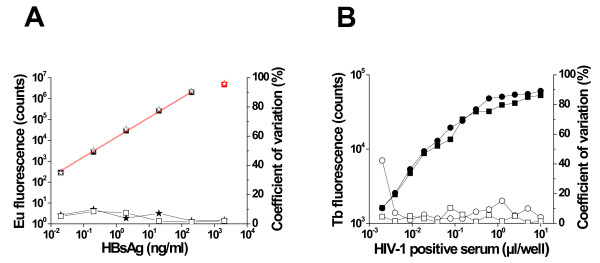
**Comparison of the sensitivity of analyte detection in single versus dual-label assay formats**. (A) HBsAg detection. Eu^3+ ^fluorescence data for the single label and dual-label assays are shown by the empty star and filled square symbols, respectively. Corresponding coefficients of variation for the single and dual-label assays are represented by the filled star and empty square symbols, respectively. (B) Anti-HIV-1 antibody detection. Tb^3+ ^fluorescence data for the single label and dual-label assays are shown by filled circles and squares, respectively. Corresponding coefficients of variation for the single and dual-label assays are represented by the empty circles and squares, respectively.

Prior to deploying the dual-label assay for the simultaneous detection of both HIV-1 and HBV infections, we evaluated its sensitivity to detect each of the two analytes (anti-HIV-1 antibody and HBsAg) in the absence (single-label) and presence (dual-label) of the binders of the other analyte. Using rHBsAg (subtype *adw*), ranging from 0.02-200 ng/mL, the dual-label assay was performed in the absence and presence of the anti-HIV-1 antibody binders, r-Bio-HIV-1 env and Tb^3+ ^chelate labeled r-HIV-1 env. Unlike in the case of rHBsAg, it is not possible to use 'known' concentrations of anti-HIV-1 antibodies, given their polyclonal nature and inherent differences in affinity and specificity for HIV-1 antigens. Thus, to explore the sensitivity of detection of anti-HIV-1 antibodies, the dual-label assay was performed using serial dilutions (as a surrogate for a range of known concentrations) of an anti-HIV-1 antibody-containing serum sample in the absence and presence of the HBsAg binders, Bio-mAb 21B and 5 S F(ab)_2 _coated Eu^3+ ^nanoparticles. The data shown in Figure 3 reveal that there was very good correlation between the single and dual-label formats of the assay with respect to each of the two analytes tested. There was essentially no discernible difference in the lowest limits of detection (LLOD) of either analyte when the two assay formats were compared. For HBsAg, the LLOD was 0.011 and 0.013 ng/mL, respectively, in the absence and presence of anti-HIV-1 antibody binders. The corresponding LLOD for anti-HIV-1 antibody detection cannot be designated for the reason mentioned above. Nevertheless, it is evident from Figure 3B that antibodies present in as low as 0.01 to 0.001 μl of the HIV-1 positive serum (used in this experiment) are detected in this assay, which reaches saturation at 1 μl of this serum. Overall, the data justify the conclusion that combining the anti-HIV-1 antibody- and HBsAg-binders in a dual-label assay will not compromise the sensitivity of detection of either analyte. This is further borne out by the analysis of sera that contain HBsAg as well as anti-HIV-1 antibodies (see below).

Next, we tested the feasibility of the dual-label assay for detecting HIV and HBV infections in human serum samples. First, we used an in-house panel of 100 'normal' serum samples that were confirmed to be negative for both HIV and HBV infections (HIV^-^/HBV^-^), using Vidas HIV Duo Quick and HBsAg Ultra kits (bioMérieux SA, Marcy I'Etoile, France). The mean Tb^3+ ^and Eu^3+ ^fluorescence readouts of these normal serum samples plus 5× standard deviation (SD) of the corresponding means were used as the cut-offs for anti-HIV antibodies and HBsAg determinations, respectively. Next, we tested a set of 37 serum samples (Department of Virology, University of Turku). These represented infected samples of which 25 were HBV^+ ^and 12 HIV^+^, using the Vidas commercial assays mentioned above. For a given analyte, signal/cut-off (S/Co) ratios <1 and ≥1 were considered as negative and positive, respectively. The results are summarized in Table 1. An analysis of these serum samples using the dual-label assay showed that while all 12 HIV^+ ^serum samples were identified to contain anti-HIV-1 antibodies, HBsAg antigen could be detected in 23 of the 25 HBV^+ ^serum samples. Of the two remaining HBV^+ ^serum samples, one was a borderline sample (see Additional file 1: Figure S1). These two serum samples tested negative for HBsAg using the single label assay also (data not shown), suggesting that the dual-label assay format *per se *did not compromise sensitivity of HBsAg detection.

**Table 1:** Evaluation of the dual-label TRF assay for simultaneous detection of HIV-1 and HBV infections

Grp	n	Infection profile (Ref assay)^a^	Dual-label assay (HIV-1^+^/HBV^+^)^b^
In-house sera panel			

1	25	HIV-1^-^/HBV^+^	0/23^c^

2	12	HIV-1^+^/HBV^-^	12/0

BBI co-infection panel			

3	13	HIV-1^+^/HBV^+^	13/10^d^

4	6	HIV-1^-^/HBV^+^	0/6

5	3	HIV-1^+^/HBV^-^	3/0

6	1	HIV-1^-^/HBV^-^	0/0

^a ^The Reference assays for the in-house sera panel were Vidas HIV Duo Quick and HBsAg Ultra assays, for anti-HIV-1 antibody and HBsAg detection, respectively; the Reference assays for the BBI co-infection panel are mentioned in the Supplementary Information (see Additional file 1). The "+" and "-" superscripts indicate positive and negative tests, respectively.

^b ^This column indicates the results obtained using the dual-label assay described in the text. The numbers shown indicate the serum samples that scored positive for both analytes in the dual-label assay.

^c^missed two HBsAg^+ ^serum samples

^d^missed three HBsAg^+ ^serum samples

To examine the performance of the dual-label assay in the background of other infections, we tested it against a BBI viral co-infection panel PCA 201 (from Boston Biomedica Inc., now SeraCare Life Sciences Inc., Milford MA). This panel was characterized for HIV-1, HBV, HCV and HTLV infections using standard commercially available reference tests (see Additional file 1: Table S1). Twenty-three of the 25 panel members were available for this study. One member of this panel was seronegative for both HIV-1 and HBV infections (sample# 24). The dual-label assay identified this correctly as HIV^-^/HBV^-^. Of the remaining 22 serum samples, 16 and 19 samples were designated as HIV^+ ^and HBV^+^, respectively, with 13 samples seropositive for both HIV-1 and HBV (Table 1). Out of these 13 HIV^+^/HBV^+ ^serum samples, 6 were positive for HCV, and two for HTLV as well. The remaining three HIV^+ ^serum samples were negative for HBV but positive for HCV and HTLV. The dual-label assay could identify 16 out of 16 HIV^+ ^serum samples (100%). It is noteworthy that one borderline serum sample (sample# 20, S/Co ratio = 1.1) was also picked up unambiguously by the dual-label test (S/Co ratio = 14.8). This essentially is indicative of enhanced sensitivity of the dual-label test and is in agreement with the conclusions based on Figure 3B. Our data show that the dual-label assay is capable of identifying HIV^+ ^serum samples regardless of the presence or absence of HBV, HCV and HTLV co-infections, with high sensitivity and specificity. However, it is to be noted that we have used r-HIV-1env as the antigen to detect anti-HIV-1 antibodies to obtain a technical proof-of-concept. Of the 19 HBV^+ ^serum samples, 13 samples were also HIV^+^, as mentioned already, and the rest (n = 6) were HIV^-^. Many of these serum samples were co-infected with HCV, HTLV or both. The dual-label assay identified 16 of the 19 HBV^+ ^serum samples, regardless of HIV, HCV or HTLV infection status. Of the 3 HBV^+ ^serum samples that were missed by the dual-label assay, one was a borderline sample (sample# 9, S/Co ratio = 1). As with the in-house serum samples, these 3 members of panel PCA 201 also turned out to be false-negative in the single label HBV assay. This rules out the possibility that Tb^3+ ^cross-talk may have masked Eu^3+ ^signals and interfered with HBsAg detection. The data show that the concordance of the dual-label assay with regard to HBsAg detection using the reference assay (Abbott EIA) is 84%. This presumably stems from low sensitivity of the mAbs used for detection of HBsAg, despite the use of a tracer F(ab)_2_-Eu^3+ ^nanoparticle for the detection of this analyte in the dual-label assay.

In conclusion, we have developed a lanthanide fluorescent reporter-based dual-label assay for the simultaneous detection of HIV-1 and HBV infections in donated blood samples. The high sensitivity of this approach derives from the temporal resolution of the long lifetime high intensity fluorescence of Eu^3+ ^and Tb^3+ ^lanthanide tracers measured by TRF. Qdots have emerged recently as highly efficient fluorescent probes. However, these have short-lived fluorescence. Therefore, TRF cannot be employed to measure their signals and their detection is limited by autofluorecence. Further, the Eu^3+ ^and Tb^3+ ^tracers used in this study are inherently fluorescent, obviating the need for additional signal development steps as in the DELFIA and LANFIA methods [2-5], and can be measured directly from the dry surface of the microtiter wells. The simultaneous detection of two analytes combined with a relatively simpler assay format eliminating the extra signal development step, will contribute to both cost and time saving.

To our knowledge, this work, which represents the first report of a dual-label HIV/HBV assay, demonstrates in principle, the feasibility of developing a multiplex assay for screening samples for multiple infections in a blood bank setting. However, a limitation is the potential for interference among the reporters, as illustrated by the Tb^3+ ^cross-talk in Eu^3+ ^measurements in this study. It may be a challenging task to eliminate the interference arising out of cross-talk among multiple fluorescent reporters. One way of circumventing the cross-talk problem in multiplexing would be to design an array-in well strategy in which multiple analyte-capturing reagents are spatially isolated in the same well and used in conjunction with a single reporter, for example Eu^3+ ^nanoparticles. In this set up, analytes can be identified based on the specific locations from which signals are detected.

## Competing interests

The authors declare that they have no competing interests.

## Authors' contributions

TM and SMT performed experiments. RV collected and characterized the human sera samples. SS and NK designed the HIV antigen and generated the monoclonal antibodies. TS, NK and KP conceived and designed the experiments. SS, TS, NK and KP interpreted the data and prepared the manuscript. All authors read and approved the manuscript.

## Supplementary Material

Additional file 1 **Myyrylainen et al (Addl files)**. The file is organized into three sections. Section 1 describes essential Methods. Section 2 provides S/Co data on the evaluation of in-house sera panel using the dual-label TRF assay (Figure S1). Section 3 provides S/Co data on the evaluation of the BBI viral co-infection panel PCA 201 using the dual-label TRF assay (Table S1) Click here for file
