# Supplementary material for: Simultaneous detection of Human Immunodeficiency Virus 1 and Hepatitis B virus infections using a dual-label time-resolved fluorometric assay
Source: J Nanobiotechnology. 2010 Nov 26;8:27. doi: 10.1186/1477-3155-8-27 (PMC3001693; doi:10.1186/1477-3155-8-27)
Supplement: Additional file 1 — Myyrylainen et al (Addl files). The file is organized into three sections. Section 1 describes essential Methods. Section 2 provides S/Co data on the evaluation of in-house sera panel using the dual-label TRF assay (Figure S1). Section 3 provides S/Co data on the evaluation of the BBI viral co-infection panel PCA 201 using the dual-label TRF assay (Table S1) [file 1477-3155-8-27-S1.DOC]

**Additional Data Files**

### Section 1: Methods

### Preparation of labeled reagents

Anti-HBsAg mAb 21B (unpublished) and rHBsAg (subtype *adw*, HyTest Ltd., Turku, Finland) were biotinylated with 40-fold and 20-fold molar excess of biotin isothiocyanate (Department of Biotechnology, University of Turku), respectively. The reaction was performed in 50 mmol/l sodium carbonate buffer, pH 9.8, at room temperature for 4 h. Biotinylated protein was segregated from free biotin by passing the reaction mixture sequentially through NAP-5 and NAP-10 columns (GE Healthcare, Sweden) using TSA buffer, pH 7.5, (50 mmol/l Tris, 150 mmol/l NaCl, 0.5 g/l NaN_3_) as eluent.

Amino groups of r-Bio-HIV-1env and r-HIV-1env (unpublished) were labeled with a 40-fold and 25-fold molar excess, respectively, of Tb^3+^ chelate (2,2′2″,2′″-{{6,6′-{4″-[2-(4-isothiocyanatophenyl) ethyl]-1H-pyrazole-1″,3″-diyl}bis(pyridine)-2,2′-diyl}bis(methylenenitrilo)] tetrakis(acetato)terbium (Department of Biotechnology, University of Turku) respectively, in 50 mmol/l sodium carbonate buffer, pH 9.8, for 16–20 hours at +4°C. The labeled proteins were separated from excess free label by passing the reaction mixture four times through NAP columns using 25 mmol/l Tris, pH 9.5, 0.9% NaCl as eluent.

5S F(ab)_2_ fragments were produced by enzymatic fragmentation of 5S mAb. Final concentration of reagents in fragmentation buffer (50 mmol/l Tris-HCl pH 7.0, 2 mmol/l EDTA) was as follows: 1 mg/ml 5S mAb, 0.2 U/ml bromelain (Sigma) and 1 mmol/l L-cysteine (Sigma). After 4 h incubation at +37^o^C, the reaction was stopped by adding 0.1 volume of freshly made N-ethylmaleimide solution (0.1 mol/l). The fragmentation product was purified on HiTrap Protein G HP chromatography column (GE Heathcare). Amino groups of 5S F(ab)_2_ were covalently coupled to activated carboxyl groups of Eu^3+^ chelate-doped Fluoro-Max^TM^ polystyrene nanoparticles (107 nm in diameter, >30,000 chelates per particle), purchased from Seradyn Inc. (Indianapolis, IN). Activation was performed using *N*-Hydroxysuccinimide (NHS) and *N*-(3-dimethylaminopropyl)-*N*-ethylcarbodiimide (EDAC), obtained from Fluka (Buchs, Switzerland). NHS and EDAC were incubated with 1.2x10^12^ nanoparticles in 10 mmol/l sodium phosphate buffer, pH 7.0, at final concentration of 9 and 1.2 mmol/l, respectively, at room temperature (RT) for 15 minutes. Activated particles were washed twice with coupling buffer (20 mmol/l MES, pH 6.1) using Nanosep Omega 300 kDa filters (Pall Corp., Ann Arbor, MI) and resuspended in coupling buffer with Labsonic U tip sonicator (B. Braun, Melsungen, Germany). 5S F(ab)_2_ (300 µg) was added to the activated particles, and incubated for 2 h at RT. Residual free active groups and unoccupied surface areas were blocked by incubating the Eu(III) nanoparticles overnight in 15 mmol/l Tris-HCl, pH 9.0, 0.1% Brij35 and 0.1% BSA at RT. The nanoparticles were washed with 2 mmol/l Tris-HCl, pH 9.0 buffer containing 0.01% Tween-20, and finally stored in the wash buffer supplemented with 0.1% BSA and 0.01% sodium azide, at +4^o^C, protected from light. Before use, the particles were mixed thoroughly and sonicated to disperse large aggregates if any.

- 1. **Determination of cross-talk between the fluorophores**

To measure Eu^3+^ nanoparticle cross-talk, 100 ng of *in vitro* biotinylated rHBsAg was added to SA-coated wells in 50 µl assay buffer (50 mmol/l sodium carbonate buffer, pH 9.6, 25 mmol/l NaCl, 0.1% Tween-20, 0.1% Triton-X 100, 2.5% BSA, 5% D-trehalose, 0.06% bovine γ-globulin, 0.05% NaN_3_, 0.01% native mouse IgG, 0.005% denatured mouse IgG, 0.2 mmol/l CaCl_2_ and 0.02 mmol/l DTPA) and incubated for 1 h at RT with slow shaking. The wells were washed four times, followed by the addition of a dilution series of 5S F(ab)_2_ coated Eu^3+^ nanoparticles (1x10^6^–1x10^9^ pcs/well) in 50 µl assay buffer. After a further hour of incubation, the wells were washed 6 times prior to measurement of Eu^3+^ and Tb^3+^ fluorescence. To measure Tb^3+^ chelate cross-talk, a dilution series of Tb^3+^ labeled r-Bio-HIV-1env (3–800 ng in 50 µl assay buffer per well) was added to SA-coated wells, incubated 1 h at RT with slow shaking, and washed six times prior to fluorescence measurement. Eu^3+^ (modified standard europium protocol; measurement height 5 mm) and Tb^3+^ (modified standard terbium protocol; measurement height 5 mm, light integrator capacitors 3, light integrator ref. level 150, flash energy area high, flash energy level 255) was measured from the surface-bound bio-conjugates in a time-resolved mode with Victor Multilabel counter.

- 1. **Dual-label TRF assay for detecting HIV-1 and HBV infections in serum samples**

Bio-mAb 21B and r-Bio-HIV-1env (each at 150 ng/well) were added to SA-coated normal capacity low-fluorescent microtiter plates (Kaivogen, Turku, Finland) in 50 µl assay buffer per well and incubated at RT with gentle shaking for 1 hour. After washing twice with the wash buffer (50 mmol/l potassium phosphate, pH 7.2, 150 mmol/l NaCl, 0.1% Tween-20, 0.5 M KCl), 10 µl serum sample and 40 µl assay buffer were added to the wells and incubated for a further 1 hour at RT. The wells were washed four times, followed by the addition of 50 ng Tb^3+^ chelate-labeled r-HIV-1env and 1x10^8^ 5S F(ab)_2_ coated Eu(III) nanaoparticles in 50 µl assay buffer. After 1 hour of incubation at RT, wells were washed six times and the fluorescence of Eu^3+^ and Tb^3+^ read as described above.

**Section 2: Figures**

Figure *S1*: Evaluation of in-house panel of HIV^+^ and HBV^+^ human serum samples using the dual label TRF assay. Panels A and B depict the S/Co values based on Eu^3+^ and Tb^3+^ signals, respectively. The dotted line indicates S/Co =1. Values above and below this are designated as positive and negative, respectively.

**Section S3: Tables**

Table *S1*: Evaluation of the Dual Label TRF assay using BBI Viral Co-infection Performance Panel (PCA 201)

| **Member**  **ID#** | **HCV**^a^ | **Hepatitis B**^a^ | | **HTLV**^a^ | | **HIV-1**^a^ | | **HBsAg Eu^b^** | **HIV Tb^c^** |
| --- | --- | --- | --- | --- | --- | --- | --- | --- | --- |
|  |  | **a** | **b** | **c** | **d** | **e** | **f** |  |  |
| 1 | + | 53.4 | + | + | P | 0.2 | - | 367.0(+) | 0.3(-) |
| 2 | - | 51.6 | + | - | - | 13.5 | P | 886.6(+) | 188.9(+) |
| 3 | + | 49.3 | + | + | P | 0.1 | - | 200.1(+) | 0.2(-) |
| 4 | - | 26.4 | + | + | P | 0.1 | - | ND | ND |
| 5 | + | 0.4 | + | + | P | 13.5 | P | -1.8(-) | 144.3(+) |
| 6 | - | 0.5 | - | - | - | 0.1 | - | ND | ND |
| 7 | + | 0.7 | - | + | P | 13.5 | P | -0.1(-) | 166.3(+) |
| 8 | + | 38.9 | + | - | - | 13.5 | P | 750.9(+) | 206.7(+) |
| 9 | + | 1.0 | - | + | P | 13.5 | P | 0.1(-) | 178.7(+) |
| 10 | - | 42.8 | + | - | - | 13.5 | P | 840.9(+) | 130.0(+) |
| 11 | + | 48.6 | + | + | P | 0.1 | - | 1.5(+) | 0.2(-) |
| 12 | + | 38.7 | + | - | - | 13.5 | P | 154.9(+) | 79.0(+) |
| 13 | - | 33.6 | + | - | - | 13.5 | P | 1489.3(+) | 54.2(+) |
| 14 | - | 51.8 | + | + | P | 0.32 | - | 368.4(+) | 0.3(-) |
| 15 | + | 64.5 | + | + | P | 0.2 | - | 124.4(+) | 0.4(-) |
| 16 | + | 0.5 | + | + | P | 13.5 | P | -1.4(-) | 152.3(+) |
| 17 | + | 42.3 | + | - | - | 13.5 | P | 83.6(+) | 176.7(+) |
| 18 | + | 5.1 | - | + | P | 13.5 | P | 0.6(-) | 21.1(+) |
| 19 | - | 45.6 | + | - | - | 13.5 | P | 1004.7(+) | 123.4(+) |
| 20 | - | 12.2 | + | - | - | 1.1 | P | 0.2(-) | 14.8(+) |
| 21 | - | 42.2 | + | - | - | 13.5 | P | 922.0(+) | 164.6(+) |
| 22 | + | 41.5 | + | - | - | 13.5 | P | 833.3(+) | 174.7(+) |
| 23 | + | 46 | + | + | P | 0.2 | - | 160.0(+) | 0.3(-) |
| 24 | - | 0.5 | - | - | - | 0.1 | - | 0.3(-) | 0.2(-) |
| 25 | - | 50.1 | + | - | - | 13.5 | P | 592.7(+) | 90.0(+) |

Table *S1* Footnotes

^a^ Assays performed using commercial kits. HBs (columns a), HTLV (columns c) and HIV-1 (columns e) assays were done with EIA kits from Abbott. In each of these instances, assays were done twice by BBI (columns a, c, & e). Other EIA kits used were from Organon Teknika (for HBc, column b) and Ortho (for HCV). Results are expressed as ‘signal-to-cut-off’(S/Co) ratios, provided by the panel supplier using the indicated commercial kits. S/Co values ≥ 1.0 are considered as positive (+) and values <1 are considered as negative (-). HTLV (column d) and HIV-1 (column f) assays were also done in blot format using kits from Genetic Systems and Dupont, respectively. ‘P’ and ‘-’ indicate the presence and absence, respectively, of antigen bands in the blot assays (columns d & f).

^b^Values indicate ‘signal-to-cut-off’ ratios obtained for HBsAg europium signals using the in-house dual label immunofluorometric assay. ^c^Values indicate ‘signal-to-cut-off’ ratios obtained for HIV terbium signals using the in-house dual label immunofluorometric assay. The results using the in-house assay are indicated in parentheses. Samples with S/Co values < 1.0 are designated as negative (-) and those with values ≥ 1.0 are designated as positive (+). The results are indicated in parentheses. ‘ND’ indicates ‘not determined’ due to lack of sample.
